# Supplementary material for: HIVprotI: an integrated web based platform for prediction and design of HIV proteins inhibitors
Source: J Cheminform. 2018 Mar 9;10:12. doi: 10.1186/s13321-018-0266-y (PMC5845081; doi:10.1186/s13321-018-0266-y)
Supplement: Supplementary file 2 — Additional file 2. Source code of HIVProtI web server. [file 13321_2018_266_MOESM2_ESM.zip › HIVprotI_Source-code/hivprotI/weka-3-6-6/doc/weka/core/logging/FileLogger.html]

FileLogger


---


|  |  |  |
| --- | --- | --- |
| **Overview** \| **Package** \| **Class** \| **Tree** \| **Deprecated** \| **Index** \| **Help** \| **Weka's home** | |  |
| **PREV CLASS**   **NEXT CLASS** | **FRAMES**    **NO FRAMES**     **All Classes** |  |
| SUMMARY: NESTED \| FIELD \| CONSTR \| METHOD | DETAIL: FIELD \| CONSTR \| METHOD |  |


---


## weka.core.logging Class FileLogger

```
java.lang.Object
  weka.core.logging.Logger
      weka.core.logging.ConsoleLogger
          weka.core.logging.FileLogger
```

**All Implemented Interfaces:**: RevisionHandler

**Direct Known Subclasses:**: OutputLogger

---

``` public class FileLogger extends ConsoleLogger ```

A simple file logger, that just logs to a single file. Deletes the file
when an object gets instantiated.

**Version:**
:   $Revision: 7462 $

**Author:**
:   fracpete (fracpete at waikato dot ac dot nz)

---

**Nested Class Summary**

**Nested classes/interfaces inherited from class weka.core.logging.Logger**

`Logger.Level`


**Field Summary**

**Fields inherited from class weka.core.logging.Logger**

`PROPERTIES_FILE`


**Constructor Summary**

`FileLogger()`


| **Method Summary** | |
| --- | --- |
| `java.lang.String` | `getRevision()`             Returns the revision string. |

**Methods inherited from class weka.core.logging.Logger**

`getMinLevel, getSingleton, log, log`

**Methods inherited from class java.lang.Object**

`equals, getClass, hashCode, notify, notifyAll, toString, wait, wait, wait`

**Constructor Detail**

### FileLogger

```
public FileLogger()
```


**Method Detail**

### getRevision

```
public java.lang.String getRevision()
```

:   Returns the revision string.

    :   **Specified by:**: `getRevision` in interface `RevisionHandler` **Overrides:**: `getRevision` in class `ConsoleLogger`
    :   **Returns:**: the revision


---


|  |  |  |
| --- | --- | --- |
| **Overview** \| **Package** \| **Class** \| **Tree** \| **Deprecated** \| **Index** \| **Help** \| **Weka's home** | |  |
| **PREV CLASS**   **NEXT CLASS** | **FRAMES**    **NO FRAMES**     **All Classes** |  |
| SUMMARY: NESTED \| FIELD \| CONSTR \| METHOD | DETAIL: FIELD \| CONSTR \| METHOD |  |


---
